# Short-term retinoic acid treatment sustains pluripotency and suppresses differentiation of human induced pluripotent stem cells

**DOI:** 10.1038/s41419-017-0028-1

**Published:** 2018-01-05

**Authors:** Maria Teresa De Angelis, Elvira Immacolata Parrotta, Gianluca Santamaria, Giovanni Cuda

**Affiliations:** 1 Department of Experimental and Clinical Medicine, Stem Cell Laboratory, Research Center of Advanced Biochemistry and Molecular Biology, University “Magna Graecia” of Catanzaro, Viale Europa, 88100 Catanzaro, Italy; 2 CIS (Centro Interdisciplinare Servizi), University “Magna Graecia” of Catanzaro, Viale Europa, 88100 Catanzaro, Italy

## Abstract

Human pluripotent stem cells (hPSCs), including human embryonic stem cells (hESCs) derived from blastocyst and human induced pluripotent stem cells (hiPSCs) generated from somatic cells by ectopic expression of defined transcriptional factors, have both the ability to self-renew and to differentiate into all cell types. Here we explored the two antagonistic effects of retinoic acid (RA) on hiPSCs. Although RA has been widely described as a pharmacological agent with a critical role in initiating differentiation of pluripotent stem cells, we demonstrate that short-term RA exposure not only antagonizes cell differentiation and sustains pluripotency of hiPSCs, but it also boosts and improves their properties and characteristics. To shed light on the mechanistic insights involved in the resistance to differentiation of hiPSCs cultured in RA conditions, as well as their improved pluripotency state, we focused our attention on the Wnt pathway. Our findings show that RA inhibits the Wnt canonical pathway and positively modulates the Akt/mTOR signaling, explaining why such perturbations, under our experimental conditions, do not lead to hiPSCs differentiation. Altogether, these data uncover a novel role for RA in favouring the maintenance of ground-state pluripotency, supporting its bivalent role, dose- and time-dependent, for hiPSCs differentiation and self-renewal processes.

## Introduction

Human embryonic stem cells (hESCs), derived from the inner cell mass (ICM) of blastocyst and human induced pluripotent stem cells (hiPSCs), generated by direct reprogramming of somatic cells, have the capacity for unlimited self-renewal and the potential to differentiate into all three primary germ layers^1^. These properties make hESCs and hiPSCs robust cell sources to understand normal development and disease, regulation of “stemness” and differentiation processes. Although the transcriptional network of pluripotency has been widely described and characterized^2^, several intrinsic and extrinsic mechanisms affecting the fine balance between undifferentiated and differentiated state need to be further investigated. Retinoids, including Vitamin A and its derivatives, are involved in embryonic development and differentiation. Several groups have demonstrated that retinoids support self-renewal of murine embryonic stem cells (mESCs) by activating the phosphatidylinositol-3-kinase (PI3K) signaling pathway and by increasing the expression of *Nanog* and *Oct4*, the critical transcription factors for the maintenance of pluripotency^3,4^. The effect of all-trans retinoic acid (RA) seems to be time- and concentration-dependent: at high concentration it induces differentiation of pluripotent stem cells (PSCs) toward pancreatic and neural cell lineages^5,6^, while short exposure of mESCs to RA during the early stages of differentiation prevents lineage entry and commitment by the synergistic action of Wnt molecules and Leukemia Inhibitory Factor (LIF)^7^. Undifferentiated secondary colonies of mESCs emerge in culture during continuous RA exposure at low concentration, resulting in the upregulation of the *Zscan4* gene, which has the peculiarity to be a marker of ESC subpopulation with high-level of pluripotency metastate^8^. Although the effects of RA signaling during high pluripotency metastate fluctuation have been described in mESCs^9^, its role in hPSCs remains not fully understood. Here, we evaluated the effects of short exposure (24 h) to RA (0.5 µM) on two independent hiPSC lines, one derived from human skin fibroblasts (hiPSCs-F) and one generated from T-Lymphocytes (hiPSCs-TL), by analyzing different sets of standard pluripotency characterization criteria, such as self-renewal and differentiation properties, proliferation, and telomere elongation. hiPSCs undergone to RA treatment acquired a boosted pluripotency state compared to those that were not treated and used as control hiPSCs. To identify the mechanisms that might be involved in the ability of hiPSCs to counteract the differentiation effect of RA, we investigated the role of the Wnt canonical pathway, which remains still controversial in hPSCs. It was reported that RA inhibits the canonical Wnt signaling pathway, activating noncanonical Wnt pathway during differentiation of mESCs^10^, while prior studies found that Wnt/β-catenin pathway maintains hESCs in an undifferentiated and self-renewing state;^11,12^ conversely, others have reported that this signaling leads to differentiation of hESCs toward primitive streak and definitive endoderm lineages^13,14^. More recently, it has been demonstrated that endogenous Wnt/β-catenin signaling is inactive in undifferentiated hESCs and it is not required for self-renewal of hESCs. Particularly, activation of Wnt/β-catenin signaling results in loss of self-renewal and induction of mesoderm lineage genes^15^.

## Materials and methods

### Cell provision

T-Lymphocytes and skin fibroblasts were obtained from two distinct subjects after achieving informed consent in a study approved by the Local Ethics Committee.

### Cell culture and chemical treatment

The hiPSCs generated from T-Lymphocytes and skin fibroblasts were routinely cultured on Matrigel-coated dishes (BD Biosciences) and maintained in mTeSR1 medium (STEMCELL Technologies, Vancouver, Canada) at 37 °C and 5% (v/v) CO_2_. Medium was changed daily and cells were passaged every 4–6 days (80% confluency) as clumps using Gentle Cell Dissociation Reagent (STEMCELL Technologies).

To establish the best RA concentration that keeps hiPSCs in an undifferentiated state, we performed titration experiments in which three different concentrations (0.5, 1.5, and 4.5 µM) of RA (Sigma Aldrich) were tested for 24, 48, and 72 h, 2 days after hiPSCs passaging. The concentration of 0.5 µM RA was chosen since it resulted the best condition in terms of morphological characteristics of treated hiPSCs (compact and flat colonies with well defined edges) and direct alkaline phosphatase (AP) activity, analyzed using the NBT/BCIP substrate solution (Thermo Fisher Scientific), accordingly to the manufacturer’s guidelines. In all our experiments, as standard RA treatment, we used a final concentration of 0.5 µM of RA diluted directly in the culture media and kept for 24 h. In addition, cells were treated with 5 μM XAV939 (Tocris), a Wnt pathway inhibitor, added 2 days after cell plating for 72 h in the absence and presence of RA.

### RNA isolation, Reverse transcription PCR (RT-PCR), and quantitative real-time PCR (qRT-PCR)

Total RNA was extracted using RNeasy Mini Kit (Qiagen) accordingly to the manufacturer’s instructions and 1 μg was transcribed using the High-Capacity cDNA Reverse Transcription kit (Applied Biosystems). Quantitative real-time PCR (qRT-PCR) was performed with Power SYBR Green PCR Master Mix (Applied Biosystems) with 25 ng of cDNA per reaction. Relative gene expression was assessed in three technical replicates of three independent biological samples and results were normalized to the glyceraldehyde-3-phosphate dehydrogenase (*GAPDH*) by using the comparative cycles of threshold (*Ct*) method (ΔΔCt). qRT-PCR assays were run on a StepOnePlus real-time PCR system (Applied Biosystems) and the data were collected and processed using StepOne software v2.3. A list of primer sequences is provided in Table 1.

**Table 1:** List of primers used for qRT-PCR

Gene	Forward primer sequence (5'-3')	Reverse primer sequence (5'-3')
*AXIN2*	AGTGTGAGGTCCACGGAAAC	CTTCACACTGCGATGCATTT
*c-MYC*	AGAAATGTCCTGAGCAATCACC	AAGGTTGTGAGGTTGCATTTGA
*c-MYC* Tg	TAACTGACTAGCAGGCTTGTCG	TCC ACATACAGTCCTGGATGATGATG
*DNMT3B*	AAGTAAACCTAGCTCGGCGAT	TTGAGATGCCTGGTGTCTCC
*GAPDH*	TCCTCTGACTTCAACAGCGA	GGGTCTTACTCCTTGGAGGC
*GATA4*	GGCCTGTCATCTCACTACGG	ATGGCCAGACATCGCACT
*HAND1*	CCAGCTACATCGCCTACCTG	CCGGTGCGTCCTTTAATCCT
*KLF4* Tg	TTCCTGCATGCCAGAGGAGCCC	AATGTATCGAAGGTGCTCAA
KOS Tg	ATGCACCGCTACGACGTGAGCGC	ACCTTGACAATCCTGATGTGG
*MESP1*	GTGCTGGCTCTGTTGGAGA	CAGAGACGGCGTCAGTTGT
*NANOG*	TGCAAGAACTCTCCAACATCCT	ATTGCTATTCTTCGGCCAGTT
*NESTIN*	CAGCGTTGGAACAGAGGTTGG	TGGCACAGGTGTCTCAAGGGTAG
*NEUROD1*	GGCTATATAACCTGAGCGCCC	ACACTCGTCTGTCCAGCTTG
*OCT4*	GGAGGAAGCTGACAACAATGAA	GGCCTGCACGAGGGTTT
*REX1*	GTGTGAACAGAACAGAAGAGGC	CTGGTGTCTTGTCTTTGCCC
SeV Tg	GGATCACTAGGTGATATCGAGC	ACCAGACAAGAGTTTAAGAGATATGTATC
*SOX17*	ACGCCGAGTTGAGCAAGA	GCGGCCGGTACTTGTAGTT
*SOX2*	GGGAAATGGGAGGGGTGCAAAAGAGG	TTGCGTGAGTGTGGATGGGATTGGTG
*TERT*	AGAAGTTCATCTCCCTGGGG	ACGTACACACTCATCAGCCA
*ZSCAN4*	GCCCAAACCACAAGAGAAGC	GCCTAGGACCGTTCTCTTCC

*Tg* transgene, *KOS* *KLF4*, *OCT4*, *SOX2,* *SeV* Sendai Virus.

### PluriTest

RNA was purified using the Absolutely MiniPrep Kit (Agilent Technologies), and processed using the Illumina TotalPrep RNA amplification Kit (Ambion, Life Technologies). cRNA was hybridized and scanned on iScan Illumina platform to the Human HT-12 v4 Expression BeadChip Kit (Illumina). Raw microarray data were uploaded to the PluriTest website (http://www.pluritest.org) and analyzed online using the published PluriTest algorithm^16,17^.

### Immunofluorescence

Cells were fixed in 4% paraformaldehyde for 30 min at room temperature followed by a washing step with phosphate buffered saline (PBS) and an incubation step for 1 h with blocking solution (3% bovine serum albumin-BSA combined with 0.1% Triton X-100 in PBS). Cells were further incubated for 3 h in blocking solution with the following primary antibodies: human Nanog (1:1000; rabbit polyclonal, PA1-097, Thermo Fisher Scientific), human Oct4 (1:400 mouse monoclonal, 60093, STEMCELL Technologies), human β-catenin (1:200 mouse monoclonal, sc-7963, Santacruz), human TRA-1-60 (1:100 mouse monoclonal, 41–1000, Thermo Fisher Scientific), human Brachyury (1:20 goat polyclonal, AF2085, R&D systems), human Sox17 (1:20 goat polyclonal, AF192, R&D systems) and human Nestin (1:1000 mouse monoclonal, 60091, STEMCELL Technologies). After several washing steps with PBS, goat anti-mouse Alexa-Fluor-647 (A-21235, Life Technologies), donkey anti-goat Alexa Fluor-594 (A-11058, Life Technologies) and goat anti-rabbit Alexa-Fluor-488 (A-11008, Life Technologies)—conjugated secondary antibodies were added for 1 h. Following incubation with secondary antibodies, cells were washed with PBS, and nuclei were counterstained with DAPI (4′-6-diamidino-2-phenylindole). Slides were mounted with Fluorescent mounting medium (Dako Cytomation). Microscopy was performed using imaging system (DMi8), filter cubes and software from Leica microsystems.

### qPCR for telomere length

To determine relative telomere length (RLT) by quantitative PCR, for each DNA sample we measured the factor by which they differed from a reference DNA (DNA from hESCs) in its ratio of telomere repeat copy number to single gene copy number. qPCR was performed as described by Cawthon^18^.

Briefly, the sample DNA (12 ng/reaction) and reference DNA (various dilutions) were extracted using GenElute Mammalian Genomic DNA Miniprep (Sigma Aldrich) and then used as templates in Power SYBR Green PCR Master Mix with specific telomere and 36B4 primers. The primer sequences were: tel 1, GGTTTTTGAGGGTGAGGGTGAGGGTGAGGGTGAGGGT; tel 2, TCCCGACTATCCCTATCCCTATCCCTATCCCTATCCCTA; 36B4u, CAGCAAGTGGGAAGGTGTAATCC; 36b4d, CCCATTCTATCATCAACGGGTACAA.

The assay was performed in triplicate and telomere (T) qPCR and single copy gene (S) qPCR were always run in separate 96-well microplates. The number of *Ct* was measured with StepOnePlus real-time PCR system (Applied Biosystems). Data were collected and processed using StepOne software v2.3. The reference DNA was used to plot the standard curve and serial dilutions of the reference DNA were prepared ranging from 100 to 1.6 ng/reaction. The standard curve for the T qPCR and the S qPCR were generated by plotting the *Ct* values of the hESCs reference sample on the *y*-axis against its respective serially diluted template DNA concentrations, ranging from 100 to 1.6 ng, on the logarithmic *x*-axis. The regression line was plotted and the equation of the straight line used to compute the relative standard *Ct* value for a template DNA concentration of 12 ng.

### Embryoid body formation

Embryoid bodies (EBs) were generated by single cell dissociation of hiPSCs-F and hiPSCs-TL by Accutase (Gibco). Single hiPS cells were cultured in ultra-low attachment plate (Corning) with mTeSR1 medium supplemented with 10 μM of the Rho-kinase inhibitor Y-27632 (Selleckchem) for 3 days to allow cell aggregation. Medium was then replaced with DMEM/F12 containing 20% knockout serum replacement (KSR), 2 mM L-glutamine, 1 × 10^−4^ M nonessential amino acids, 1 × 10^−4^ M 2-mercaptoethanol, and 0.5% penicillin and streptomycin. The medium was changed every other day until day 8^1^. After 8 days in culture as floating EBs, cell aggregates were transferred on to 0.1% gelatin-coated plates and cultured in the same medium for additional 8 days before collecting the EBs for immunofluorescence and qRT-PCR analysis.

Bright field images of EBs were captured using an imaging system (DMi8) inverted optical microscope at ×5 magnification. EBs diameters were manually assigned and compiled by using ImageJ software. For irregularly shaped EBs, the longest diameter was analyzed. The data set was then exported to Microsoft Excel for data analysis.

### Cell proliferation assay

hiPSCs (-F and -TL) were treated with CellTrace CFSE (cell proliferation kit, Thermo Fisher Scientific) at a final concentration of 8 μM for 10 min at 37 °C. Labeling was blocked by the addition of cold PBS with 0.1% BSA and incubated for 5 min on ice. Two hours later (day 0) and after 5 days of culture in standard medium or RA condition, the fluorescence intensity was measured by flow cytometry (BD LSRFortessa x-20). Cell proliferation was calculated by monitoring the decrease in label intensity in successive daughter cell generations^19^. The proliferation index and the cell populations of parental or different generations were calculated by Modfit LT Version 3.2 software.

### Western blot

Whole-cell lysate extracts were prepared with RIPA Buffer (Sigma Aldrich) supplemented with protease inhibitor and phosphatase inhibitor cocktail (Thermo Fisher Scientific). The protein content was determined by Bradford analysis (Bio-Rad). Equal amounts of proteins (30–50 μg) were separated on 4–15% Mini-PROTEAN TGX Precast Protein Gels (Bio-Rad) and transferred onto nitrocellulose membrane (Bio-Rad). After blocking in TBS-T (0.05% Tween 20) containing 5% nonfat dry milk for 1 h at room temperature, the membrane was incubated overnight at 4 °C with primary antibodies against Nanog (PA1-097, Thermo Fisher Scientific), Oct4 (60093, STEMCELL Technologies), β-catenin (sc-7963, Santacruz), phospho-Akt Ser473 (9271, Cell Signalling), phospho-Akt Thr308 (4056, Cell Signalling), Akt1 (2967, Cell Signalling), mTOR (2983, Cell Signalling), phospho-mTOR Ser2448 (5536, Cell Signalling), S6K1 (2708, Cell Signalling), phospho-S6K1 Thr389 (9206, Cell Signalling), and Actin (sc-1616, Santa Cruz).

The membrane was then washed three times with TBS-T, incubated with a 1:10,000 dilution of horseradish peroxidase-linked secondary antibodies (Jackson ImmunoResearch), and the immunoreactive proteins were visualized with the enhanced chemiluminescence detection system (Bio-Rad).

### Gene set enrichment analysis

To determine the biological impact of RA treatment, we performed a Gene Set Enrichment Analysis (GSEA), which is a computational method that predicts whether an a priori defined set of genes shows statistically significant enrichment for a particular biological state. A first comparison was ran between RA_8_ treated and untreated hiPS cell lines. Furthermore, to assess statistical significance, we randomized our data set by permuting gene sets 1000 times and considered only gene sets with a *p* ≤ 0.01. GSEA was conducted using an algorithm implemented in R software^20^.

### Statistical analysis

All experiments were performed at least three times. Data were analyzed using the GraphPad Prism 6 software and statistical analysis was performed by Student’s *t*-test. All values were expressed as mean ± standard error of the mean (SEM) in all figure panels in which error bars are shown and differences with *p < 0.05, **p < 0.01, ***p < 0.001 were considered statistically significant.

## Results

### Short-term RA treatment maintains hiPSCs in an undifferentiated state

**Fig. 1:**
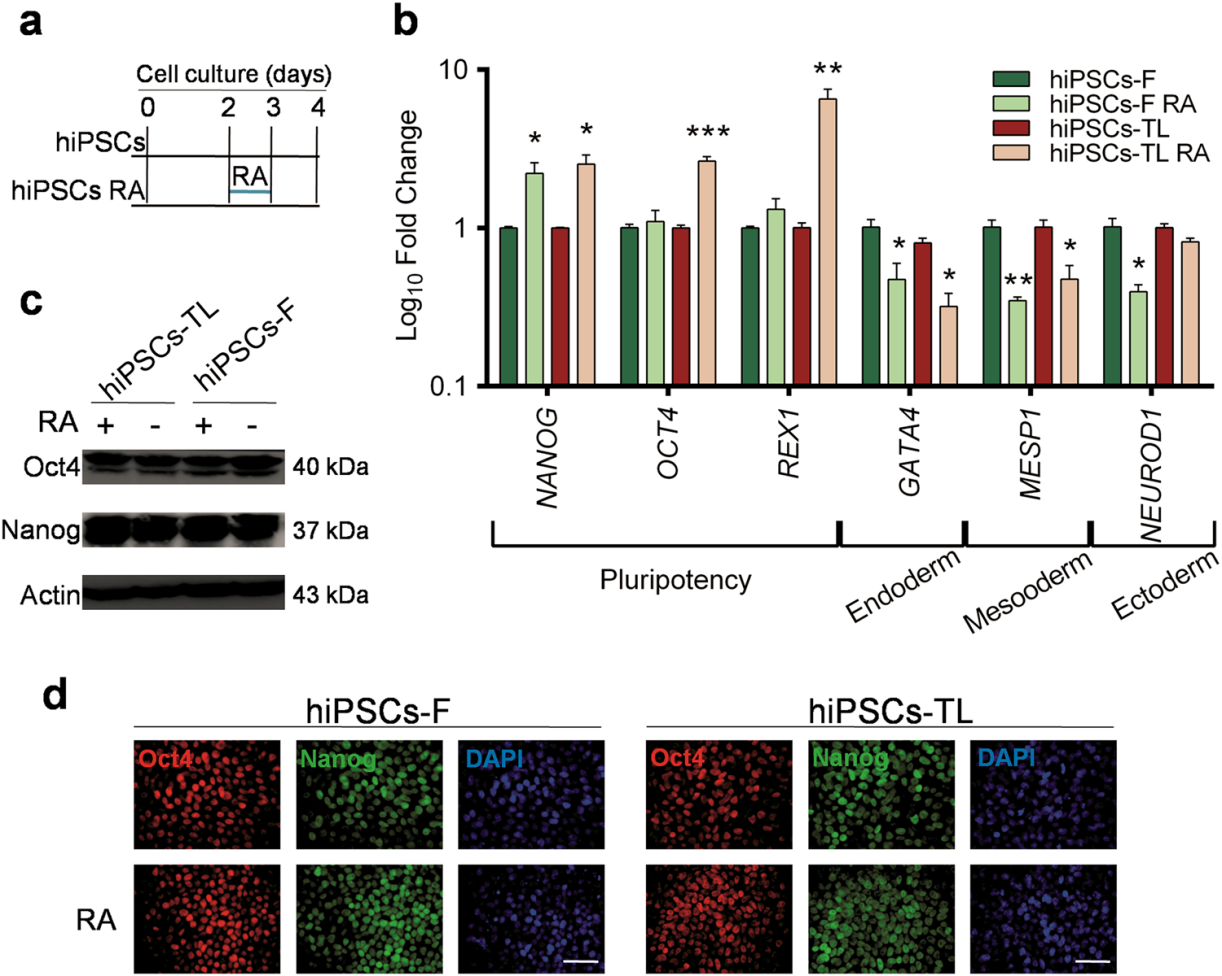
Effects of short-term retinoic acid treatment on pluripotency **a** hiPSCs were cultured in regular medium (mTeSR1) for 5 days before ready for passaging, while hiPSCs-RA were treated with RA for 24 h 2 days after splitting. **b** qRT-PCR analysis of pluripotency and differentiation markers in hiPSCs (-F and -TL) cultured in regular medium and in medium supplemented with 0.5 µM RA at day 4. All expression values are normalized to *GAPDH* and relative to untreated hiPSCs. Data are mean ± SEM from three independent experiments. A statistical comparison was made between hiPSCs-RA and hiPSCs by Student’s *t*-test (*p < 0.05, **p < 0.01, ***p < 0.001). **c** Western-blot analysis of Oct4 and Nanog for hiPSCs (-TL and -F) cultured with (+) or without (−) 0.5 µM RA. Actin was used as the loading control. **d** Immunofluorescence of Oct4 (red) and Nanog (green) in hiPSCs and hiPSCs-RA. Nuclei were counterstained with DAPI (blue). Scale bar=50 μm

RA has been widely associated with pluripotent stem cells mostly because of its role in promoting specific cell lineage commitment. Here we investigated the role of short-term (24 h) RA exposure on self-renewal and pluripotency of two independent hiPSC lines, hiPSCs-F and hiPSCs-TL. The hiPSC lines were passaged and plated at clonal density in standard maintenance medium. After 2 days, standard medium was supplemented with three different RA concentrations (0.5, 1.5, and 4.5 µM) and cells were analyzed at day 3 (24 h from RA treatment) and day 4 (24 h from RA withdrawal) (Fig. 1a). hiPSCs (-F and -TL) undergone to 0.5 µM RA treatment at day 4, showed a pluripotency state comparable to control cells cultured without RA, confirmed by the expression analysis of the pluripotency genes, with a 2-fold increase of *NANOG* expression in both cell lines, 2- and 6-fold increase of *OCT4* and *REX1* levels in hiPSCs-TL while the expression of *OCT4* and *REX1* remained basically unchanged in hiPSCs-F-RA (Fig. 1b). On the other hand, expression of lineage-specific differentiation markers (*GATA4*, *MESP1,* and *NEUROD1*) were reduced after RA exposure (Fig. 1b). These data suggest that the addition of RA at low concentration and for a short window of time positively affects the pluripotent state of hiPSCs and counteracts their differentiation.

hiPSCs exposed to higher RA concentration (1.5 and 4.5 µM, respectively) at day 3 already showed clear signs of differentiation: the edges of the colonies were less compact (Supplementary Fig. S1a) and the expression levels of the pluripotency markers *OCT4*, *NANOG*, and *REX1* gradually decreased to increasing of RA concentrations (Supplementary Fig. S1b). On the other hand, 24 h after RA (1.5 and 4.5 µM) withdrawal (day 4), the ESC-like morphology of colonies was immediately restored, as shown by AP staining (Fig. S2a). As latter effect, we noticed an overall restoration of the expression pattern of the pluripotency markers *OCT4*, *NANOG*, and *REX1*, whose levels gradually increased (Supplementary Fig. S2b). Interestingly, 0.5 µM RA treatment for 48 h or longer promoted differentiation in both cell lines (Supplementary Fig. S3), thus confirming a dose- and time-dependent effect of RA.

The effects of 0.5 µM RA treatment were further investigated at protein level: western blot analysis did not highlight any significant difference in the expression levels of the pluripotency markers Oct4 and Nanog in RA-treated vs. untreated hiPSCs (Fig. 1c), although levels of transcripts were slightly elevated in cells grown in RA medium; moreover, a homogeneous distribution and co-localization of both markers in the whole colony was observed by immunofluorescence analysis (Fig. 1d). These data reveal that short-term RA treatment consistently prevents differentiation in both PSCs, as indicated by a reduction of differentiation markers rather than a perturbation of pluripotency markers.

**Fig. 2:**
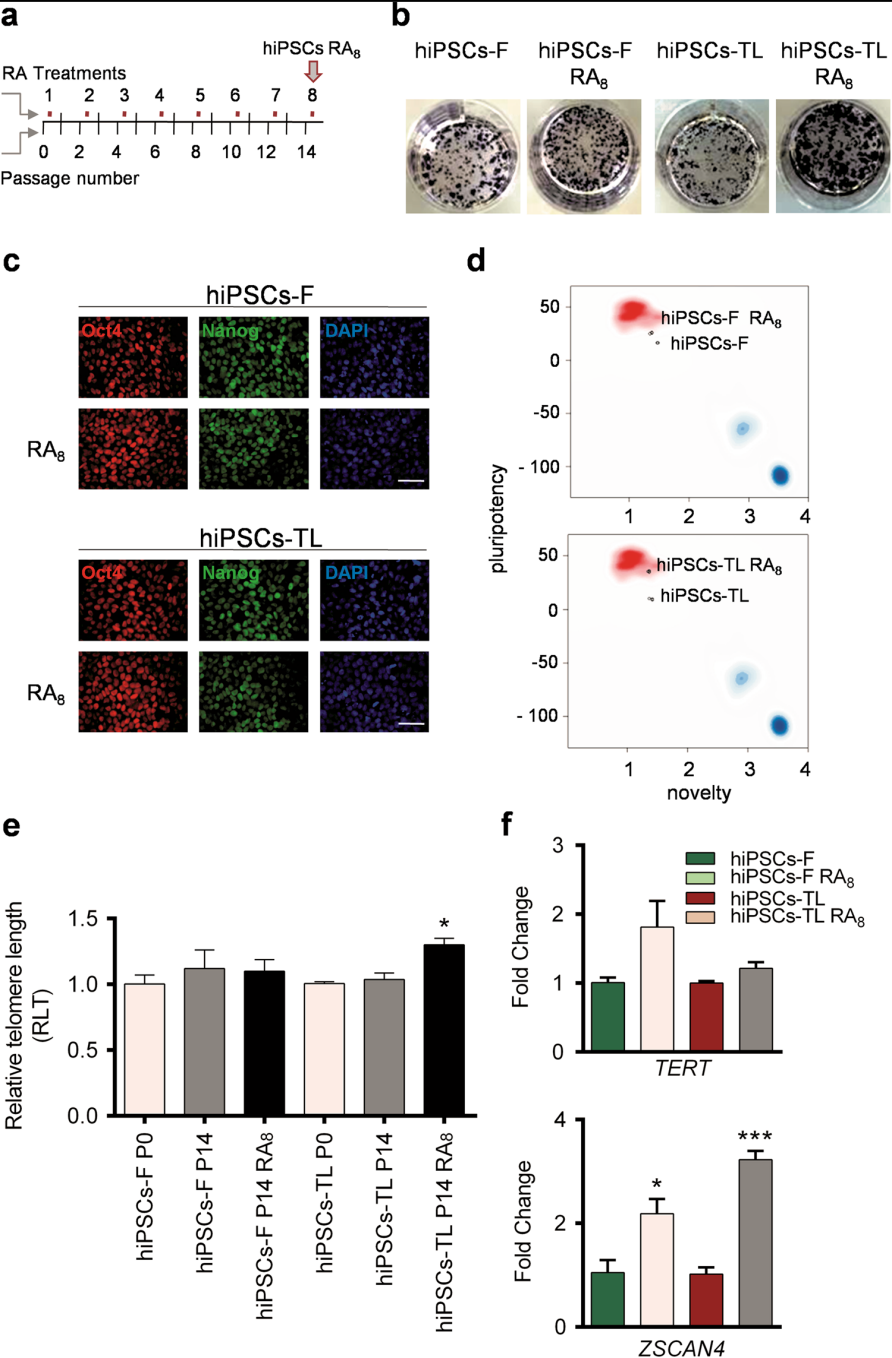
Experimental design and response of hiPSCs to RA treatment over time **a** hiPSCs were treated with 0.5 µM RA every second passage for a total of 8 steps over time during a culture period of 14 passages; cells that have undergone to 8 passages RA treatment are named hiPSCs-RA_8_. **b** Representation of AP activity in hiPSCs-RA_8_ and hiPSCs. Maintenance of pluripotency in hiPSCs-RA_8_ was confirmed by immunofluorescence for Oct4 (Red) and Nanog (Green) **c** and with depth-gene expression analysis by PluriTest algorithm **d**. **e** Quantitative real-time PCR (qPCR) of relative telomere length (RTL) assessed in hiPSCs-F and hiPSCs-TL, in three experimental conditions (regular medium, P0; regular medium P14; RA_8_ exposure P14) showing maintenance of length of telomeres during RA exposure. A statistical comparison was made between hiPSCs P14 RA_8_ and hiPSCs P0 or hiPSCs P14 RA_8_ and hiPSCs P14 by paired Student’s *t*-test (*p < 0.05). **f** qRT-PCR analysis of *TERT* and *ZSCAN4* levels in hiPSCs-RA_8_ and relative untreated cells, showing the induction of *TERT* and *ZSCAN4* expression in hiPSCs-RA_8_. All expression values are normalized to *GAPDH* and relative to untreated hiPSCs. A statistical comparison was made between hiPSCs-RA_8_ and hiPSCs by Student’s *t*-test (*p < 0.05, ***p < 0.001). Data are mean ± SEM from three independent experiments. RA_8_ = cells were treated for 8 times with retinoic acid in 14 passages. Scale bar=50 μm

To gain additional information concerning the beneficial effect of RA on self-renewal and pluripotency of hiPSCs, we performed a time-course analysis exposing the hiPSCs to 0.5 µM RA for 14 passages, during which cells were treated for 24 h with RA every second passage. The cells used in the time-course were named hiPSCs-RA_8_, and a schematic representation of the time course is shown in Fig. 2a. Under monitored and constant exposure to RA, hiPSCs continued to form standard, dome-shaped colonies with homogeneous and strong activity for AP (Fig. 2b), while the expression of pluripotency-related genes in hiPSCs-RA_8_ remained unchanged (*REX1* in hiPSCs-F) or it was slightly upregulated (*NANOG*, *OCT4* in both cell lines and *REX1* in hiPSCs-TL) with respect to untreated hiPSCs (Supplementary Fig. S4a). Western blot and immunofluorescence analysis showed comparable levels of Oct4 and Nanog in hiPSCs-RA_8_ and untreated hiPSCs (Fig. 2c and Supplementary Fig. S4b).

To further test the level of pluripotency of hiPSCs-RA_8_, we performed a PluriTest assay, which interrogates a large-scale of datasets of genome-wide pluripotent and somatic expression profile, giving a measure of the divergence of the global stem cell transcriptome from expected patterns present in well-characterized pluripotent stem cells. Interestingly, the PluriTest analysis revealed a higher Pluripotency Novelty Score in hiPSCs-RA_8_ compared to the untreated ones, indicating that hiPSCs (-F and -TL) exposed to 0.5 µM RA for 8 times during a period of 14 passages, acquired an increased level of pluripotency (Fig. 2d).

Telomere length and the increase of telomerase activity are among the most critical key factors involved in self-renewal capability, reprogramming, proliferation, and differentiation efficiency of ES cells^21^. RA treatment is known to decrease telomerase activity in breast carcinoma cells, while no effects have been shown in human oral keratinocytes^22,23^, suggesting that RA might have a tissue/cell specificity. To test if RA, under our experimental conditions, plays a role in telomere elongation, we measured telomere length at time 0 and after 14 passages under RA condition by qPCR. As shown in Fig. 2e, the telomere elongation rate remained basically unchanged in untreated hiPSCs (P0 and P14 of the time-course) of both lines and in hiPSCs-F P14 RA_8_, while it was increased in hiPSCs-TL P14 RA_8_. These data were further evaluated by qRT-PCR analysis for genes known to be involved in telomere elongation, such as telomerase reverse transcriptase subunit (*TERT*) and *ZSCAN4*, whose expression levels resulted slightly induced (*TERT*) and significantly increased (*ZSCAN4*) in hiPSCs-RA_8_ compared to hiPSCs cultured in standard conditions (Fig. 2f). The increased expression of *ZSCAN4* might be likely responsible for the differentiation resistance of RA-treated hiPSCs, as previously described^8^.

Taken together, these results confirm that telomere elongation is preserved independently of the passage and/or the number of RA treatments in hiPSCs.

### Embryoid body formation is enhanced in RA-treated hiPSCs

**Fig. 3:**
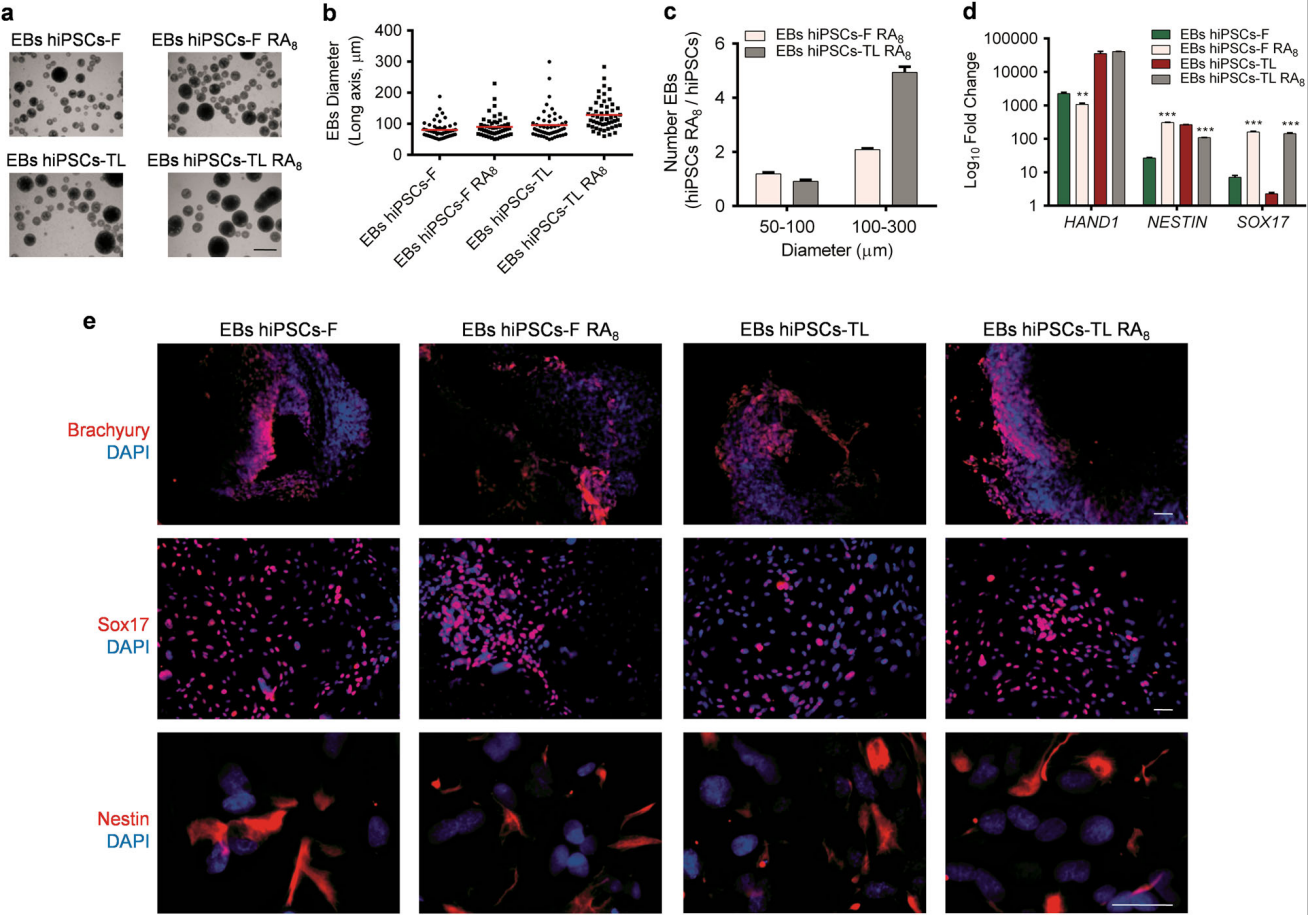
In vitro differentiation capacity of RA_8_-treated hPSCs **a** Representative image of EBs formation capability of RA-treated and untreated hiPSCs-F and -TL. Scale bar, 500 μm. **b** Diagram indicating the average diameter of the EBs, 100 μm. **c** The efficiency of EBs formation with diameter range of 100–300 µm is higher in hiPSCs-RA_8_ compared to untreated cells, while the percentage of EBs with diameter range of 50–100 μm is comparable between hiPSCs-RA_8_ and hiPSCs. **d** The potential of hiPSCs-RA_8_ to differentiate into cell of all three germ layers by expression analysis of *HAND1*, *NESTIN*, and *SOX17*, the gene expression levels in EBs hiPSCs and EBs hiPSCs-RA_8_ were relative to hiPSCs. qRT-PCR data are represented as the mean ± SEM from three independent experiments. A statistical comparison was made between EBs hiPSCs-RA_8_ and EBs hiPSCs-RA by Student’s *t*-test (**p < 0.01, ***p < 0.001). **e** Immunostaining of whole EBs for Brachyury (upper panel), Sox17 (middle panel), and Nestin (bottom panel) demonstrating the differentiation potential of hiPSCs-RA_8._ Scale bar, 50 μm

The stemness of RA-treated hiPSCs was further investigated by performing an Embryoid Body formation assay, which represents one of the crucial criteria to assess pluripotency and direct differentiation capability of hiPSCs in vitro. It is known that the size distribution of EBs plays a significant role in the efficiency of differentiation and in production yields^24,25^. hiPSCs formed regular EBs in RA conditions (Fig. 3a) and the average diameter of the EBs is about 100 μm (Fig. 3b). Therefore, we determined the differences in EBs size by measuring the diameter along their major axis. Interestingly, the efficiency of hiPSCs-F RA_8_ to form EBs with a diameter range of 100–300 µm was about ~ 2-fold higher compared to EBs derived from untreated hiPSCs-F, and about 5-fold higher for EBs derived from hiPSCs-TL RA_8_ compared to the untreated counterpart. The percentage of EBs with diameter range of 50–100 μm was comparable between hiPSCs-RA_8_ and hiPSCs (Fig. 3c). EBs generated from hiPSCs-RA_8_ showed a similar pattern of differentiation ability compared to untreated cells with regard to the expression of *HAND1*, a mesodermal marker, while the *SOX17* (endodermal marker) expression resulted upregulated in both RA_8_-hiPS cell lines; finally, *NESTIN* (ectodermal marker) was upregulated exclusively in hiPSCs-F RA_8_ (Fig. 3d). We also analyzed the expression level of the differentiation markers in EBs derived from RA-treated hiPSCs compared to EBs obtained from control, untreated cells. Even though the EBs derived from hiPSCs-RA do differentiate in all three germ layers, we did not obtain the same *SOX17* upregulation detected in EBs derived from hiPSCs-RA_8_ (Supplementary Fig. S5).

Characterization of EBs derived from hiPSCs-RA_8_ was completed by immunofluorescence staining for markers of the three germ layers, namely Brachyury (mesoderm), Sox17 and Nestin (Fig. 3e).

Overall, these data demonstrate that hiPSCs periodically exposed to RA not only retain their pluripotency state but also do not compromise their ability to differentiate toward the three germ layers, with preferential capability toward endoderm lineage in both hiPSCs.

### RA maintains hiPSCs pluripotency through inhibition of the canonical Wnt/β-catenin pathway and activation of the Akt pathway via mTOR

**Fig. 4:**
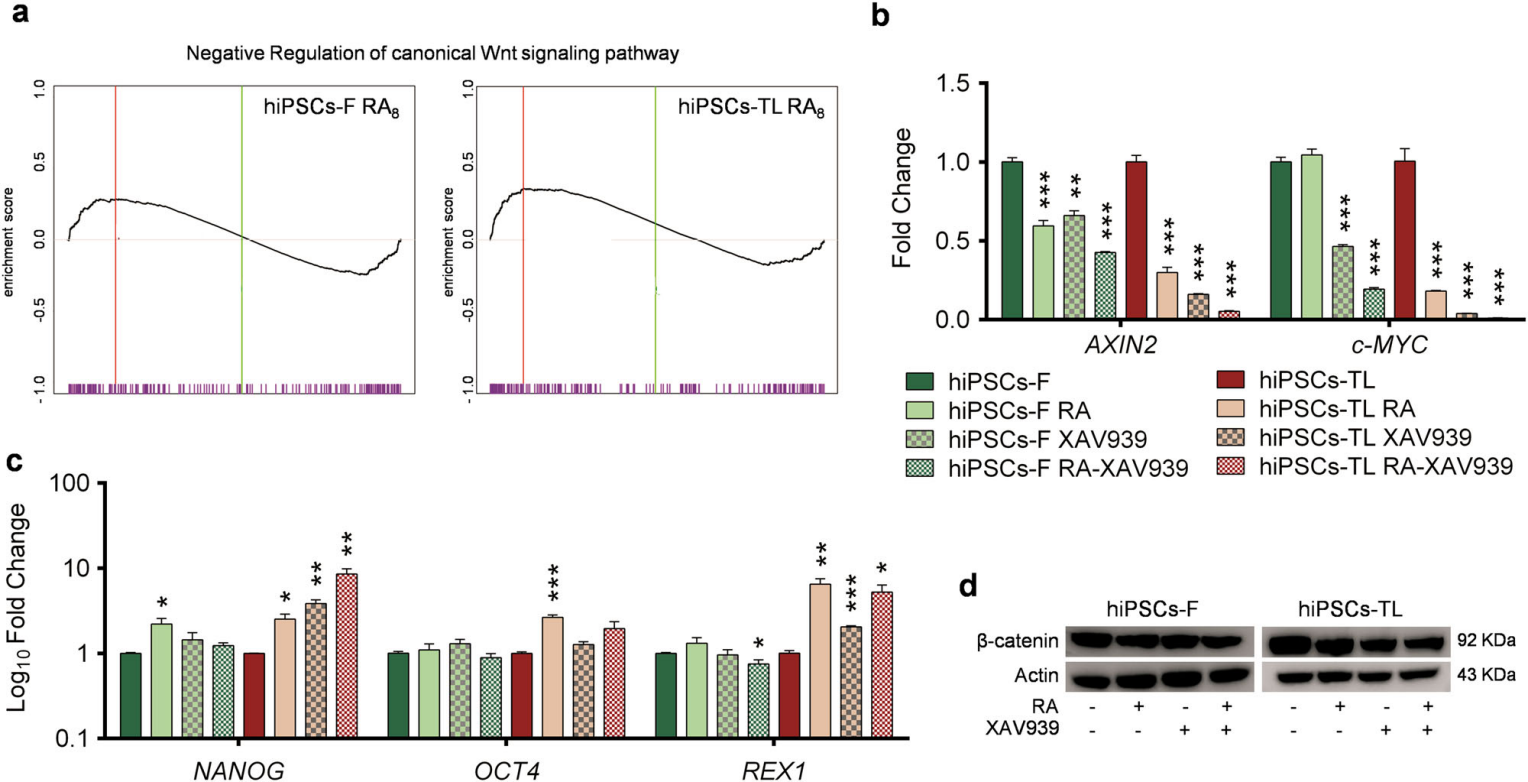
RA maintains the pluripotency of hiPSCs through the inhibition of Wnt signaling pathway **a** GSEA enrichment plot for Wnt target genes in -RA_8_ vs. untreated hiPSCs-F (*p* < 0.05, nes = 1.187), and in hiPSCs-TL-RA8 vs. untreated counterpart (*p* < 0.05, nes = 1.466). qRT-PCR analysis for *AXIN2* and *c-MYC* **b** and for *NANOG*, *OCT4*, and *REX1* **c** in hiPSCs cultured in media supplemented either with RA alone, or XAV939 alone or with a combination of both molecules (RA-XAV939) confirm the down-regulation of Wnt target genes and reveal a maintainance of expression levels of pluripotency-associated genes in hiPSCs treated compared to untreated ones. All expression values are normalized to *GAPDH* and relative to untreated hiPSCs. Data are mean ± SEM from 3 independent experiments; a statistical comparison was made between hiPSCs-RA and hiPSCs or hiPSCs-XAV939 and hiPSCs or hiPSCs-RA-XAV939 and hiPSCs by Student’s *t*-test *p < 0.05, **p < 0.01, ***p < 0.001). **d** Immunoblot analysis for β-catenin on proteins extracted from hiPSCs, hiPSCs-RA, hiPSCs-XAV939 and hiPSCs-RA-XAV939. Protein loading was normalized to equal levels of Actin. Total protein levels of β-catenin were reduced in RA, XAV939 and RA-XAV939 condition compared to control cells

To investigate the molecular mechanism(s) and downstream targets of RA, we performed transcriptome analysis of untreated and RA_8_-treated hiPSCs. Pathway analysis using the Gene Set Enrichment Analysis (GSEA) software revealed that negative regulator genes involved in the Wnt canonical pathway were significantly enriched in hiPSCs-RA_8_ compared with untreated cells (Fig. 4a).

Wnt/β-catenin activation does not appear to be able to maintain the undifferentiated and pluripotent state of hESCs^26^, and its activation has been identified as critical in the loss of self-renewal of ESCs^15^. β-catenin is a core component of the Wnt canonical pathway and, when the pathway is activated, β-catenin migrates to the nucleus where it regulates the expression of target genes such as *AXIN2* and *c-MYC*. Here, we analyzed the modulation status of Wnt/β-catenin signaling pathway in hiPSC lines after RA treatments.

qRT-PCR performed to monitor the expression levels of *AXIN2* and *c-MYC*, demonstrated a reduction of both transcripts, except for *c-MYC* in hiPSCs-F. Analogous results were obtained by treating the cells with the Wnt/β-catenin signaling inhibitor XAV939 (tankyrase inhibitor), and finally, a synergistic effect was noticed by using a combination of RA and XAV939 (Fig. 4b).

These data brought us to investigate the role of Wnt/β-catenin signaling in the maintenance of hiPSCs pluripotency. Under the same experimental conditions, hiPSCs showed an undifferentiated ESC-like morphology (data not shown) and, more interestingly, maintained unaltered the expression of the major pluripotency-associated transcriptional factors *NANOG*, *OCT4* and *REX1* (Fig. 4c).

We further extended our analysis by immunofluorescence assays for subcellular localization of β-catenin. The analysis revealed a nuclear/cytoplasmic distribution of β-catenin in hiPSCs exposed to RA, XAV939 and RA-XAV939, with a prevalent cytosolic localization of the fluorescent signal at the edges of the hiPSCs colonies. Intriguingly, under the same experimental conditions, few hiPSCs colonies did not show positivity for β-catenin staining (Supplementary Fig. S6).

The involvement of the canonical Wnt pathway was finally evaluated by western blot analysis for β-catenin, revealing a reduction in the expression level (~ 30%), which was comparable between RA, XAV939 and RA-XAV939-treated with respect to untreated hiPSCs (Fig. 4d).

**Fig. 5:**
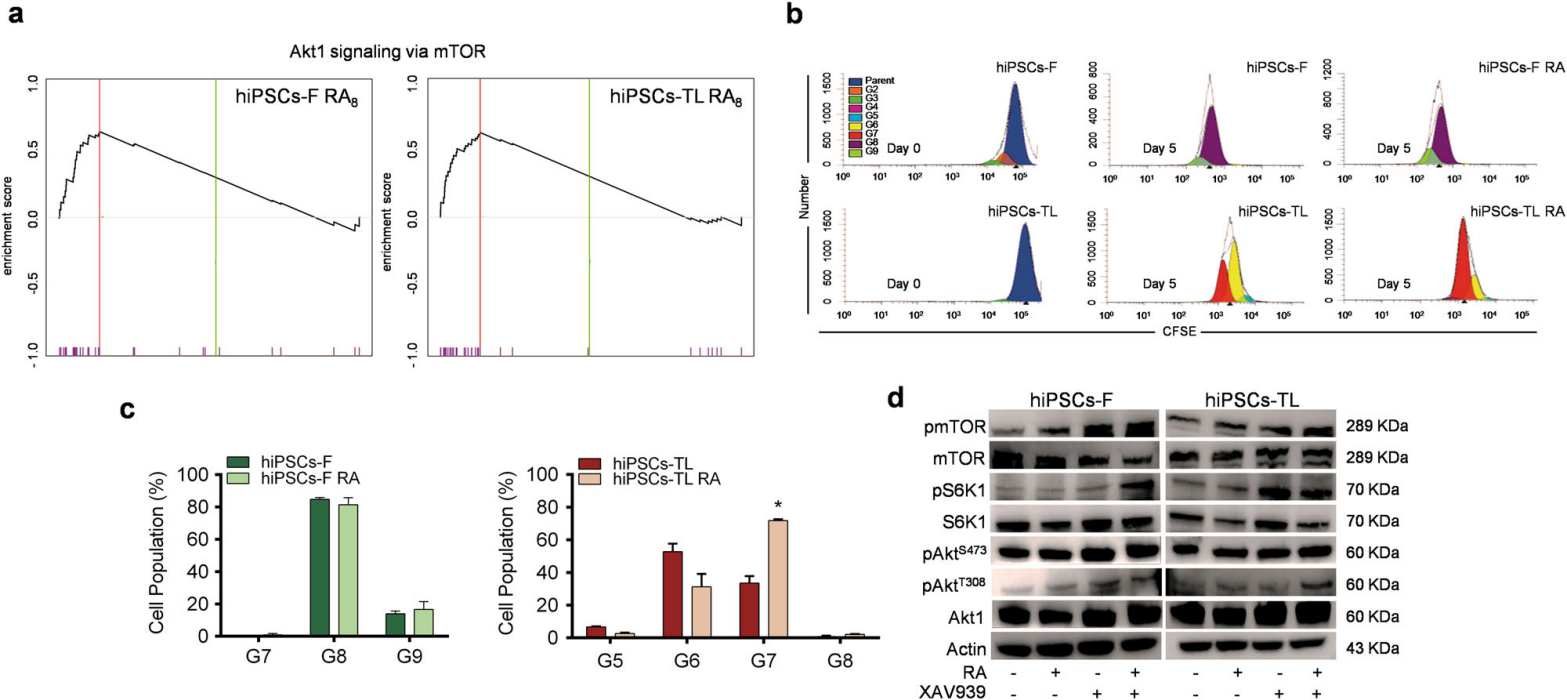
Cell proliferation assay in human iPSCs after RA exposure **a** GSEA analysis showing enrichment of Akt1/mTOR signature in RA_8_ treated cells. hiPSCs-F RA_8_ (*p* < 0.01, nes = 2.1); hiPSCs-TL RA_8_ (*p* < 0.01, nes = 2.0). **b** Representation of cell proliferation rate by flow cytometric analysis of hiPSCs-F and -TL stained with CFSE cultured with or without RA for 24 h. **c** Diagrams showing the quantification of proliferation rate relative to hiPSCs-F-RA compared to hiPSCs-F (left), and relative to hiPSCs-TL-RA compared to hiPSCs-TL (right). Quantitative data are expressed as mean ± SEM of three independent experiments. A statistical comparison was made between of hiPSCs-RA and hiPSCs for each generation by paired Student’s *t*-test (*p < 0.05). **d** Western blot analysis for mTOR, pmTOR (Ser2448), S6K1, pS6K1 (Thr389), Akt1, pAkt^S473^, and pAkt^T308^, on hiPSCs, hiPSCs-RA, hiPSCs-XAV939, and hiPSCs-RA-XAV939. An increase of pAkt^T308^, pmTOR and its downstream pS6K1 was observed in hiPSCs after treatment with RA, XAV939 or RA-XAV939 compared to control. Under the same experimental conditions, pAkt^S473^ levels were unalterated

In order to shed more light on the molecular mechanisms underlying the effects of RA treatment on pluripotency maintenance, a GSEA analysis was performed, with a specific focus on the Akt signaling pathway. This analysis highlighted a strong enrichment of Akt1 via mTOR target gene sets in hiPSCs RA_8_ compared to the untreated ones, suggesting that this pathway might be deregulated under RA condition (Fig. 5a).

Akt regulates cell growth and apoptosis by phosphorylating several proteins, such as TSC1/2 which leads, in turn, to mTOR activation^27^. To explore the RA effect mediated by Akt on hiPSCs survival, we stained the cells with a cell-tracking dye, CFSE. The fluorescence staining was distributed to successive generations of cells as shown in Fig. 5b, indicating that RA does not induce proliferation arrest of hPS cells. While the proliferation rate of hiPSCs-F did not differ significantly between treated and untreated conditions, the RA-treated hiPSCs-TL, instead, showed an increased percentage of cells in later generations (G7) compared with the untreated cells (Fig. 5c).

Akt1 is activated by site-specific phosphorylations: Thr308 and Ser473. Thr308 phosphorylation by PDK1 partially activates Akt1^28^, while the phosphorylation at Ser473 is required for full activation of Akt signaling^29^. It was reported that the upregulation of PDK1 upon activation of PPARβ/δ by RA in HaCaT cells results in increased phosphorylation of Thr308^30^. On this basis, we tested the Akt phosphorylation state in hiPSCs after RA, XAV939 and RA-XAV939 exposure and we noticed that, while pAkt^S473^ levels were substantially unaltered in comparison to untreated cells, pAkt^T308^ increased significantly in treated vs. untreated hiPSCs (Fig. 5d).

Akt phosphorylation, in turn, results in the activation of mTOR^31^ and the existence of an mTOR-dependent signaling mechanism for maintaining pluripotency and suppressing differentiation of hESCs by inhibition of the Wnt signaling pathway has been recently reported^32^. In our experimental model, RA and XAV939 enhanced mTOR activity, as indicated by increased levels of the phospho-mTOR (pmTOR), and its downstream target, ribosomal subunit S6 kinase 1 (pS6K1) (Fig. 5d).

**Fig. 6:**
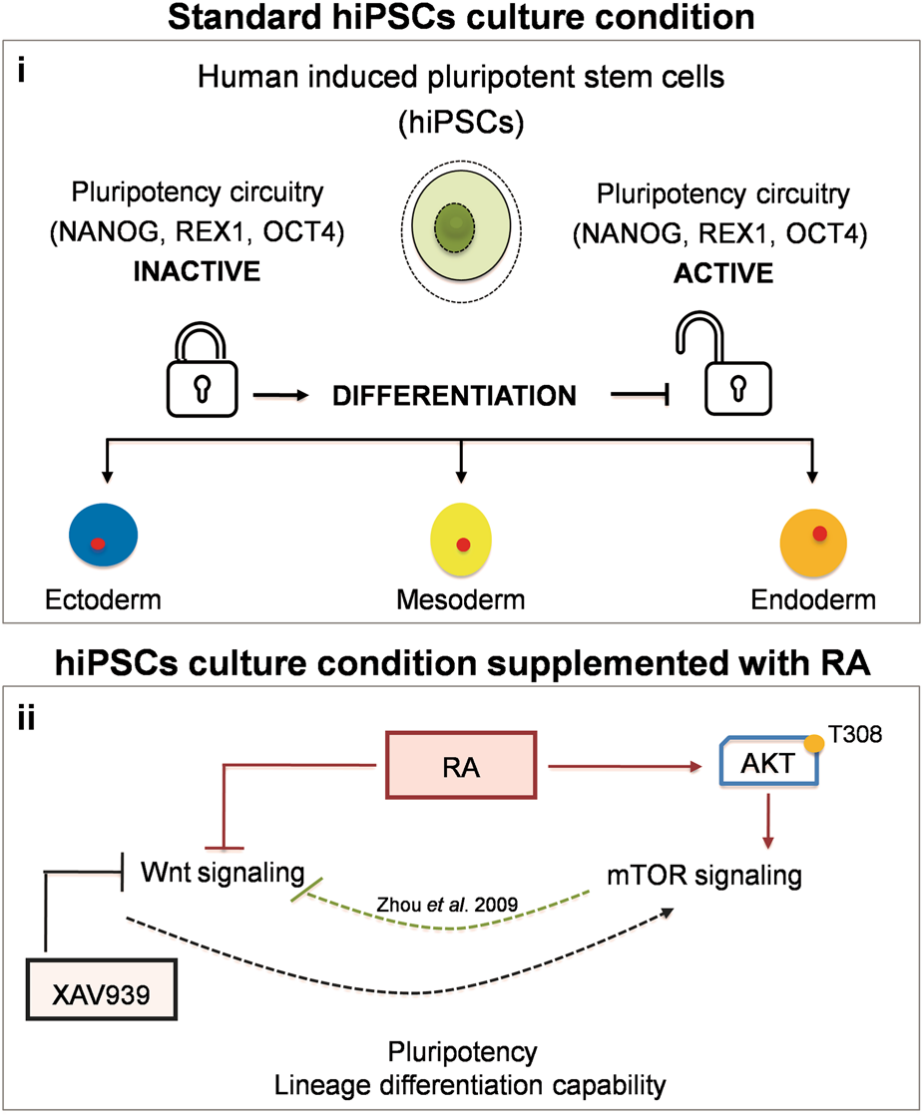
Overview of the findings presented in this study **i** Under standard culture conditions, hiPSCs show a remarkable potential to self-renew while maintaining their pluripotency when the circuitry of pluripotency network is activated; on the other hand, hiPSCs can give rise to any cell lineage when the pluripotency network is inactivated upon stimulation of differentiation. **ii** Short-term RA treatment of hiPSCs regulates pluripotency and differentiation through inhibition of the canonical Wnt pathway and activation of mTOR signaling. hiPSCs exposed to RA maintain pluripotency in culture and improve the differentiation potential

Altogether, these findings suggest a peculiar role of RA in regulating the cross-talk between Akt via mTOR and Wnt/β-catenin pathways in hiPSCs. Zhou et al. reported that mTOR represses the activity of several differentiation transcription factors partially by suppressing the Wnt signaling pathway in hESCs. On this basis, we inferred that RA is able to induce mTOR activation in hiPSCs through Akt signaling. As a consequence, the pluripotency state in hPSCs in mainly maintained by a repression of the differentiation process. A graphical representation of this model is illustrated in Fig. 6.

## Discussion

Self-renewal and differentiation are opposite mechanisms but equally important for pluripotent stem cells. How the pluripotency and self-renewal are maintained is topic of great importance for the potential clinical applications of hiPSCs, such as regenerative medicine and cell transplantation. Furthermore, the differentiation of hPSCs in vitro mimics the early embryonic development, providing opportunities to understand the cell biology of early development and disease. RA has been shown to play a critical role during early embryonic development, but opposite effects of RA have been also reported. RA counteracts differentiation of mESCs in the absence of LIF and, conversely, favors the emergence of undifferentiated colonies from differentiated cells in the presence of LIF^7,8^, suggesting that RA may participate in the regulation of pluripotency. In the present study, we demonstrate that a short-term exposure to RA (0.5 μM, 24 h) maintains hiPSCs in a pluripotent state. hiPSCs colonies treated with RA show typical ES-like morphology, positivity for AP staining, and preserved expression of pluripotency genes. The maintainance of pluripotency of hiPSCs treated with RA is a dose-dependent process, defining a fine balance between pluripotency and differentiation. We show that the mRNA and protein levels of *OCT4*, *NANOG* are unaltered after RA treatment, while the transcriptional activity of *GATA4*, *MESP1,* and *NEUROD1* is down-regulated. These results raise the possibility that RA exposure might cause rapid perturbations of differentiation markers independently of the *OCT4*, *NANOG* circuitry.

Here we demonstrate, for the first time to our knowledge, that short and transient exposure of hiPSCs to RA positively affects their pluripotency state, which results augmented when several aspects of pluripotency are investigated. RA treatment enhances the efficiency of EBs formation, as demonstrated by the fact that hiPSCs-RA_8_ generate more and bigger EBs (100–300 µm) compared to hiPSCs cultured in regular medium, with a stronger capacity to differentiate, preferentially toward the endoderm germ layer. The role of RA in enhancement of pluripotency and differentiation may be explained by the activation of the RA signaling pathway, shown to improve the generation of iPSCs^33,34^. Another beneficial effect of RA in hiPSCs is represented by the lack of telomere shortening, despite several studies have suggested that RA down-regulates the telomerase activity in certain types of cancer cells^35^. Our data show an induction of telomere elongation in hiPSCs-TL RA_8_ compared to hiPSCs-TL, supported by the overexpression of *TERT* and *ZSCAN4*, previously shown to contribute to telomere elongation^36^.

The decision of PSCs to maintain *naive* state or to differentiate can be modulated through destabilization of the pluripotency network involving, among others, the Wnt/β-catenin signaling pathway. Many reports indicate that this pathway is required to stimulate ES cell self-renewal and to maintain the pluripotent state^11,12^. Conversely, other studies have found that its activation leads to differentiation toward mesoderm and endoderm lineages and does not promote self-renewal^14,15,37^. In our study, we identify the inhibition of the canonical Wnt/β-catenin pathway as a major mechanism by which RA is capable to retain the pluripotency and counteract the spontaneous differentiation of hiPSCs, confirming results obtained in mESCs, where inhibition of β‐catenin/TCF interaction improves functional pluripotent characteristics of stem cells^38^. These data are further strengthened by results in which RA treatment is combined with hiPSCs exposure to the Wnt inhibitor small molecule, XAV939, revealing a reduction of β-catenin in response to RA or XAV939 treatment, with a significant down-regulation in the expression levels of *AXIN2* and *c-MYC* after the treatments. These results support our hypothesis that RA regulates Wnt canonical pathway in hiPS cells. Furthermore, GSEA analysis on hiPSCs-RA_8_ revealed, together with an enrichment of Wnt signaling pathway, a significant enrichement of the Akt via mTOR pathway.

Furthermore, we show that inhibition of Wnt positively influences mTOR activity; considering that XAV939 exposure mirrored the effect of RA, we hypothesize that the maintainance of pluripotency throught repression of differentiation markers of hiPSCs cultured with RA is mediated by a reduction of β-catenin secondary to the activation of mTOR triggered by an increase of pAkt^T308^.

The Akt/mTOR pathway plays a key role in preventing apoptosis^39,40^. Here we provide evidence that RA-treated cells correlate with an increasing number of colony attachment (data not shown) and the lack of proliferation arrest.

In conclusion, our data provide an innovative and highly efficient strategy for the enhancement of pluripotency of hiPSCs, which still maintain unaltered their capability to differentiate into any of the three germ layers. The novel culture approach described here and the characterization of the molecular mechanisms by which RA improves pluripotency, represent significant findings in long-term expansion of hiPSCs in a well-defined culture condition (RA_8_). Finally, we depict a novel role of RA in the context of stem cell biology, to control and affect pluripotency and the induction of differentiation, modulating negatively the Wnt pathway and positively the mTOR signaling.

## Electronic supplementary material

Figure S1

Figure S2

Figure S3

Figure S4

Figure S5

Figure S6

Figure S7

Supplementary Figure Legends
