# Supplementary figures and images for: Short-term retinoic acid treatment sustains pluripotency and suppresses differentiation of human induced pluripotent stem cells
Source: Cell Death Dis. 2018 Jan 5;9(1):6. doi: 10.1038/s41419-017-0028-1 (PMC5849042; doi:10.1038/s41419-017-0028-1)

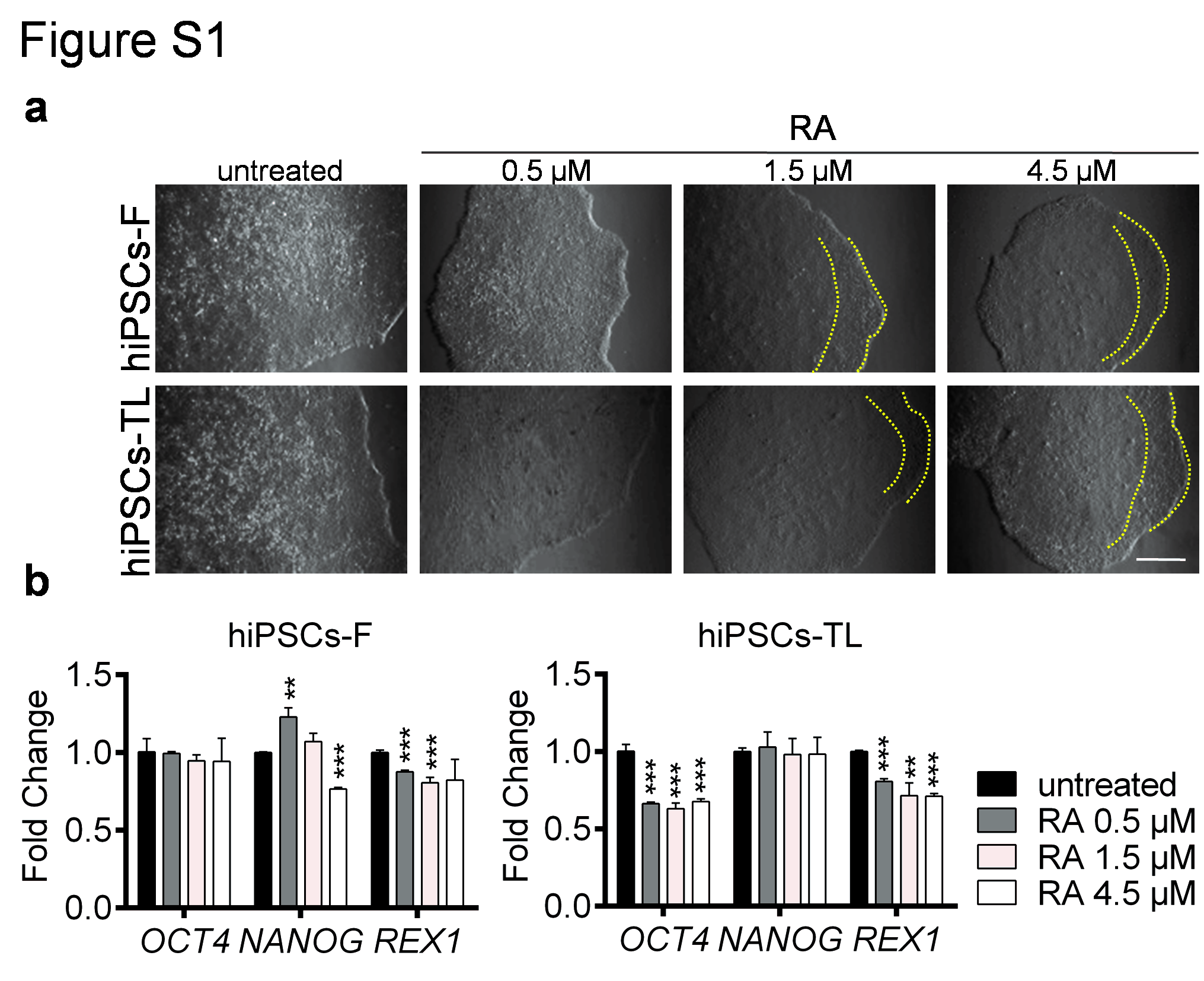

Supplement: Supplementary file 1 — Figure S1 [file 41419_2017_28_MOESM1_ESM.tif]

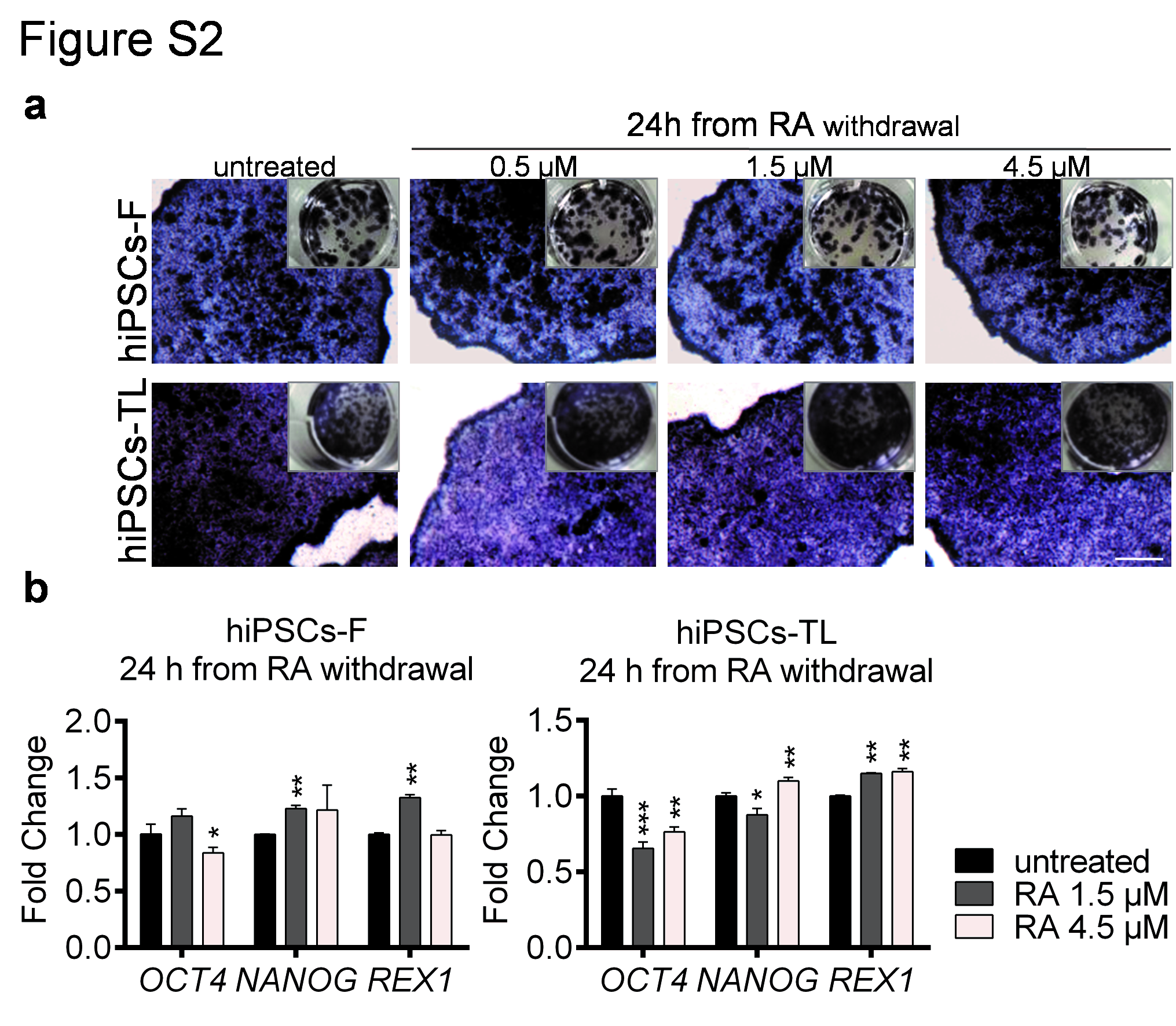

Supplement: Supplementary file 2 — Figure S2 [file 41419_2017_28_MOESM2_ESM.tif]

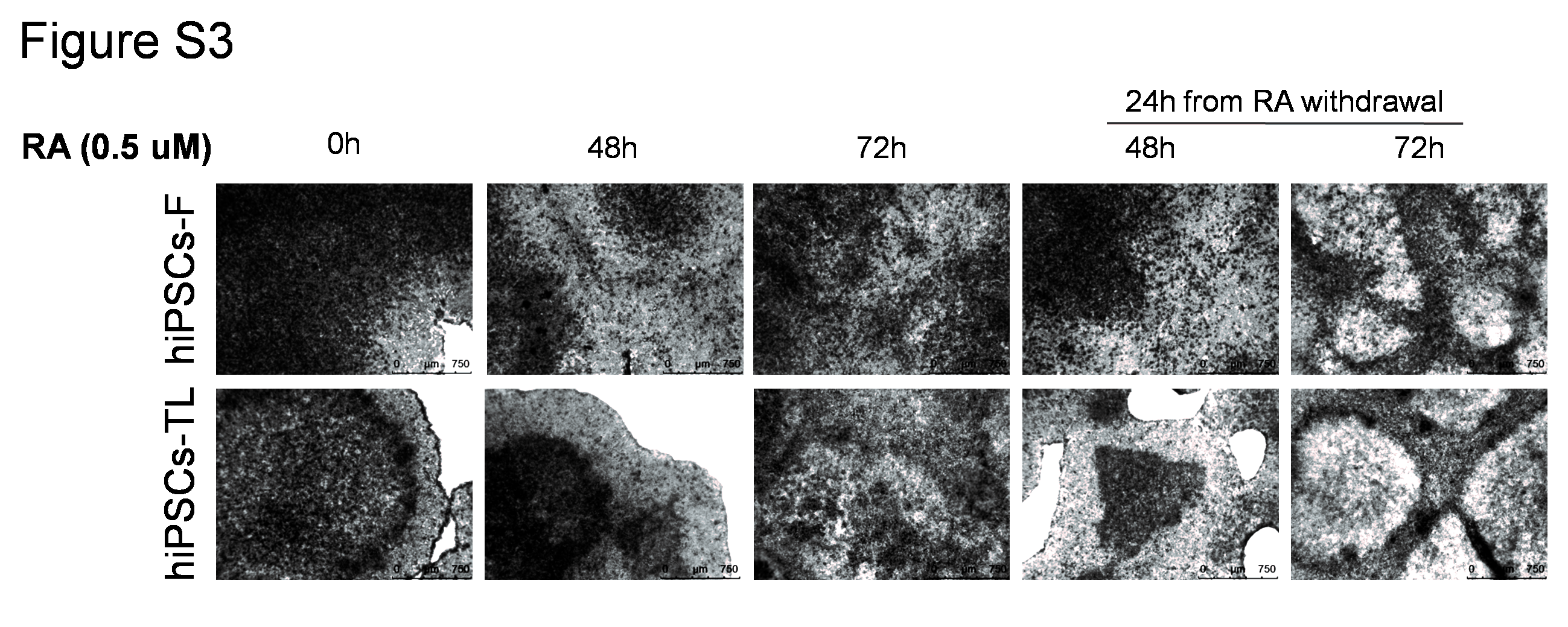

Supplement: Supplementary file 3 — Figure S3 [file 41419_2017_28_MOESM3_ESM.tif]

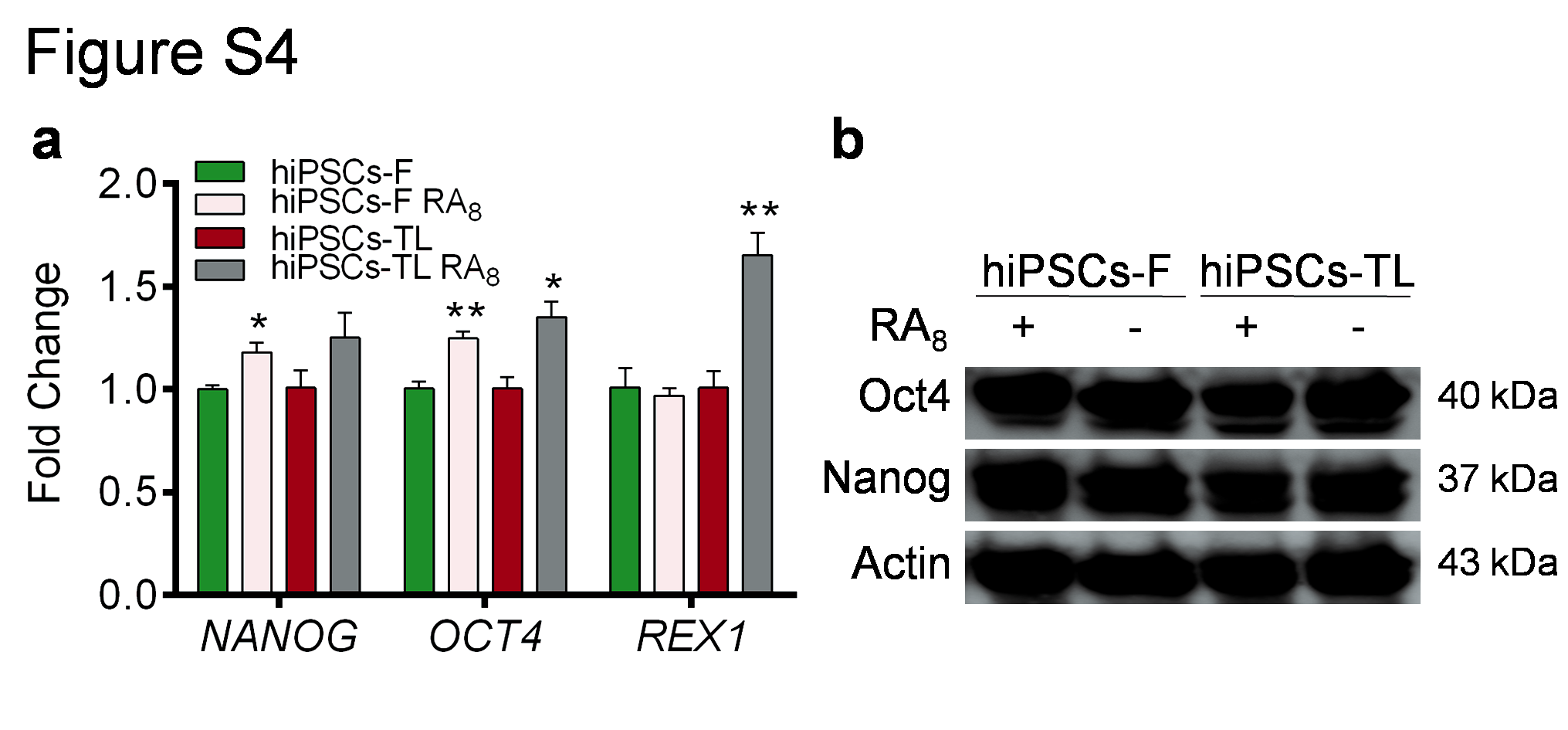

Supplement: Supplementary file 4 — Figure S4 [file 41419_2017_28_MOESM4_ESM.tif]

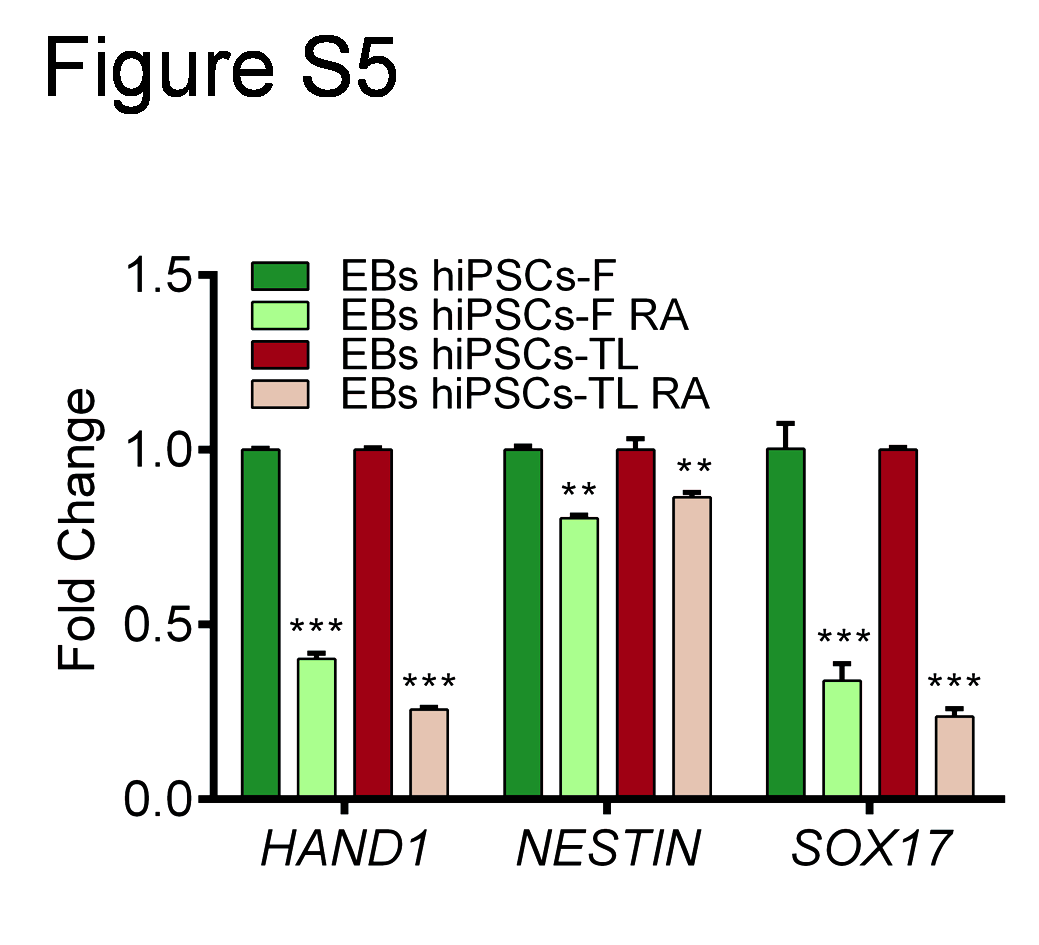

Supplement: Supplementary file 5 — Figure S5 [file 41419_2017_28_MOESM5_ESM.tif]

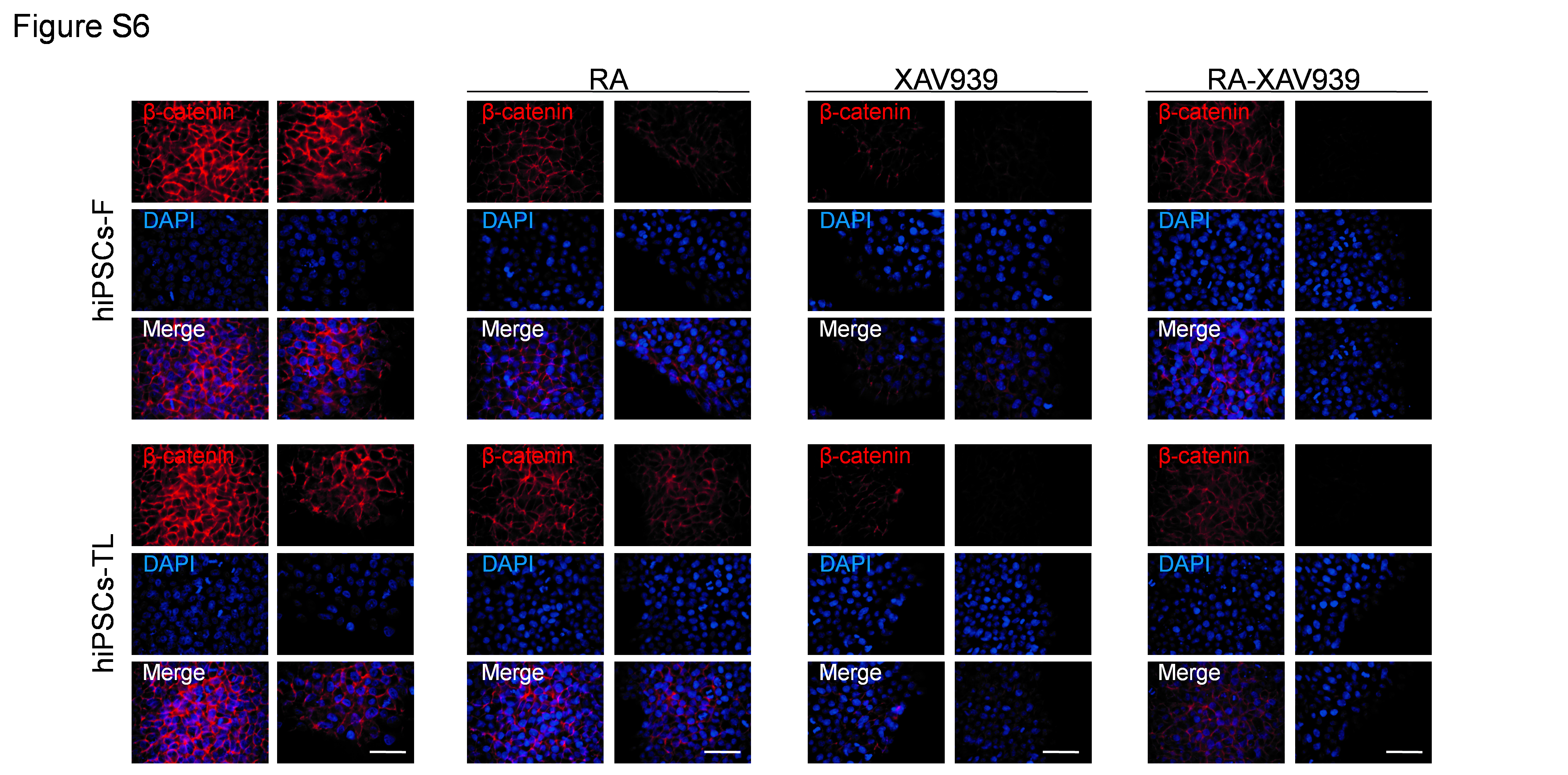

Supplement: Supplementary file 6 — Figure S6 [file 41419_2017_28_MOESM6_ESM.tif]

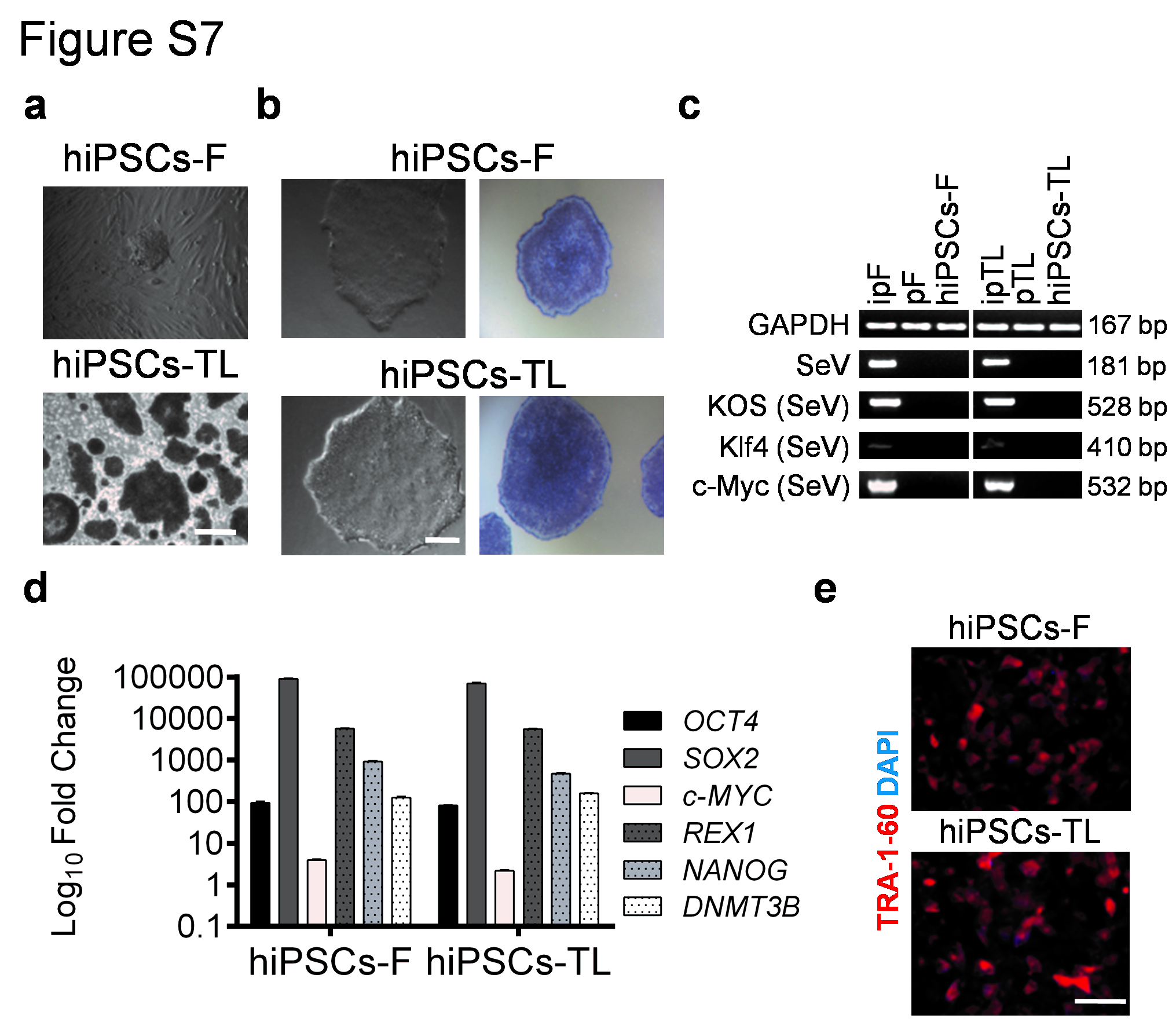

Supplement: Supplementary file 7 — Figure S7 [file 41419_2017_28_MOESM7_ESM.tif]
