# Supplementary material for: Short-term retinoic acid treatment sustains pluripotency and suppresses differentiation of human induced pluripotent stem cells
Source: Cell Death Dis. 2018 Jan 5;9(1):6. doi: 10.1038/s41419-017-0028-1 (PMC5849042; doi:10.1038/s41419-017-0028-1)
Supplement: Supplementary file 8 — Supplementary Figure Legends [file 41419_2017_28_MOESM8_ESM.docx]

**SUPPLEMENTAL DATA**

*Human iPSC generation*

Skin biopsy and peripheral blood of two healthy donors were processed for fibroblasts and T-lymphocytes isolation and expansion, respectively. Skin fibroblasts were isolated from the tissue by the outgrowth method, while peripheral blood was subjected to Ficoll Gradient (Lymphosep, Biowest) and T-lymphocytes were expanded and activated by anti-CD3 antibody (R&D systems) and IL-2 (Life Technologies) for 5 days in AIM-V medium (Life Technologies). Subsequently, 5x10^5^ T-lymphocytes and 3x10^5^ skin fibroblasts were transfected with Sendai Viruses (CytoTune, Life Technologies) encoding *OCT3/4*, *SOX2*, *KLF4*, and *c-MYC* (at MOI of 20 and 5, respectively) for iPSCs generation.^41^

Yielded hiPSCs clones were manually picked and cultured under standard protocols for feeder-free conditions on matrigel (BD Biosciences) - coated plates and mTeSR1 medium (STEMCELL Technologies) (Figure S7a).

*Characterization of induced pluripotent stem cells*

Generated iPSC lines showed ESC-like morphology, positivity for alkaline phosphatase (Figure S7b) and loss of Sendai Viral transgenes (Figure S7c).

Genome-wide gene expression profile of undifferentiated hiPSCs showed high similarity to pluripotent stem cells, as confirmed by PluriTest with a high “pluripotency score” and a low “novelty score” (Figure 2d) and showed reactivation of endogenous pluripotency genes *OCT4*, *SOX2*, *c-MYC*, *REX1*, *NANOG*, and *DNMT3B* (Figure S7d). Moreover, generated hiPSCs revealed positive immunoreactivity for the embryonic stem-cell markers Oct4, Nanog (Figure 1d) and TRA-1-60 (Figure S7e). Before performing experiments, all cell lines were tested for mycoplasma contamination.

**Supplementary Figure Legends**

**Figure S1**

Assessment of different retinoic acid concentrations.

(**a**) Representative images of the morphology acquired by hiPSCs-F and hiPSCs-TL when exposed to three different RA concentrations (0.5, 1.5 and 4.5 μM) for 24 h and relative to untreated cells. 0.5 μM RA for 24 h gave the best morphological results exhibiting defined edges, compact and homogeneous colonies. (**b**) qRT-PCR analysis of the pluripotency-associated genes *OCT4*, *NANOG* and *REX1* in hiPSCs-F and hiPSCs-TL 24 h after RA treatment at different concentrations, displays the starting down-regulation of these pluripotency genes. All expression values are normalized to *GAPDH* and relative to untreated hiPSCs. Data are mean ± SEM from 3 independent experiments; ***p* < 0.01, ****p*<0.001. Scale bar, 250 μm.

**Figure S2**

Evaluation of morphological and molecular hiPSCs changes after RA withdrawal.

(**a**) Representive images of hiPSCs-F and -TL colonies after RA (0.5, 1.5 and 4.5 μM) withdrawal, showing a restored hESC-like morphology and AP activity.

(**b**) qRT-PCR analysis for the pluripotency markers *OCT4*, *NANOG*, and *REX1* 24 h after RA (1.5 and 4.5 μM) withdrawal, reveals the renewal of the expression level of these genes. All expression values are normalized to *GAPDH* and relative to untreated hiPSCs. Data are mean ± SEM from 3 independent experiments; **p* <0.05, ***p* < 0.01, ****p*<0.001. Scale bar, 250 μm.

**Figure S3**

Time lapse of RA exposure effect on hiPSC morphology.

Representative images of the hiPSCs morphology were acquired after AP staining. hiPSCs were analysed after 48 and 72 h of 0.5 μM RA exposure and 24 h after RA withdrawal at the same time points.

**Figure S4**

Effects of repeated RA exposure on pluripotency of hiPSCs .

(**a**) qRT-PCR analysis of pluripotency genes *NANOG*, *OCT4* and *REX1* in hiPSCs-RA_8_. All expression values are normalized to *GAPDH* and relative to untreated hiPSCs. Data are mean ± SEM from 3 independent experiments; **p* < 0.05, ***p* < 0.01. (**b**) The protein levels of Nanog and Oct4 were evaluated by western blot. Actin was used as the loading control.

**Figure S5**

*In vitro* differentiation capability of RA-treated hPSCs.

qRT-PCR analysis of *HAND1*, *NESTIN*, and *SOX17* levels in EBs hiPSCs-RA and EBs hiPSCs. All expression values are normalized to *GAPDH* and relative to EBs hiPSCs. Data are represented as the mean ± SEM from 3 independent experiments; ***p* < 0.01, ****p*<0.001).

**Figure S6**

β-catenin localization in hiPSCs before and after treatments.

Immunofluorescence analysis with β-catenin (red) antibody and co-staining with DAPI (blue) was performed on hiPSCs-F and hiPSCs-TL treated with RA, XAV939 and RA-XAV939. Scale bar 50 μm.

**Figure S7**

Generation and characterization of human induced pluripotent stem cells (hiPSCs) from healthy donor T-lymphocytes and skin fibroblasts.

(**a**) Representative images of fibroblasts at day 20 after SeV-transduction (hiPSCs-F), and PBMCs at day 5 of activation in the presence of anti-CD3 antibody and IL-2 (hiPSCs-TL)**.** (**b**) Generated hiPSCs show normal hESC morphology and typical alkaline phosphatase activity. (**c**) RT-PCR confirms presence (lane 1) of Sendai viral transgenes in infected fibroblasts (ipF) and T lymphocytes (ipTL), absence in parental fibroblasts (pF) and T lymphocytes (pTL) (lane 2), and loss of transgenes in hiPSCs-F and hiPSCs-TL (lane 3). (**d**) qRT-PCR analysis of endogenous pluripotency genes *OCT4*, *SOX2*, *c-MYC*, *REX1*, *NANOG*, and *DNMT3B*. All expression values are normalized to GAPDH and relative parental cells. (**e**) Immunostaining of hiPSCs for the pluripotent stem cell marker TRA-1-60 (red) and co-staining with DAPI (blue). Scale bar 250 μm (a-b) and 50 μm (e).
